# RE-COVER project: A survey on resilience, mental health, and fear of Covid-19 in four countries

**DOI:** 10.1186/s13104-021-05819-x

**Published:** 2021-11-04

**Authors:** Daichi Sugawara, Yuan Gu, Akihiro Masuyama, Siew Li Ng, Evone Y. M. Phoo, Raja Intan Arifah Binti Raja Reza Shah, Takahiro Kubo, Yuta Chishima, Eugene Y. J. Tee

**Affiliations:** 1 Faculty of Human Sciences, University of Tsukuba, 1-1-1 Tennodai, Tsukuba, 305-8752 Japan; 2 Department of Public Administration, Humanities and Arts, Dalian Maritime University, Dalian, 116026 China; 3 Faculty of Psychology, Iryo Sosei University, Chuodai-Iino 5-5-1, Iwaki, Fukushima 970-8551 Japan; 4 Department of Psychology, HELP University, 40150 Shah Alam, Malaysia; 5 Department of Social, Health and Organisational Psychology, Utrecht University, Utrecht, Netherlands; 6 Faculty of Human Sciences, University of Tsukuba, Otsuka 3-29-1, Bunkyo-ku, Tokyo 112-0012 Japan

**Keywords:** Resilience, Mental health, Fear of COVID-19, Well-being

## Abstract

**Objectives:**

The COVID-19 pandemic has impacted the mental health of people worldwide. Psychological resilience has been shown to buffer against the threat of the pandemic (i.e., COVID-19 fear) and sustain mental health. The extent to which psychological resilience factors impact mental health maintenance, however, is unclear, given broad differences in infection rates, prevention approaches, government interventions across different cultures and contexts. Our study examines resilience factors and how they protect individuals from COVID-19-related fear and sustain their mental health.

**Data description:**

Data were collected from 1583 (M_age_ = 32.22, SD = 12.90, Range = 19–82) respondents from Japan, China, the United States, and Malaysia between October to November 2020. We collected data across age and sex, marital status, number of children, and occupations. We also accounted for stay-at-home measures, change in income, COVID-19 infection status, place of residence, and subjective social status in the study. Our variables included mental health-related and resilience constructs, namely (i) fear of COVID-19, (ii) depression, anxiety, and stress; (iii) present, past, and future life satisfaction, (iv) sense of control, (v) positive emotions, (vi) ego-resilience, (vii) grit, (viii) self-compassion, (ix) passion, and (x) relational mobility. All questionnaires were assessed for their suitability across the four countries with the necessary translation checks. Results from this study can be instrumental in examining the impact of multiple resilience factors and their interaction with demographic variables in shaping mental health outcomes.

## Objective

As of February 2021, The World Health Organization (WHO) reports that 113 million people were infected with the coronavirus disease 2019 (COVID-19), with the pandemic claiming the lives of approximately 2.5 million people worldwide [1]. The COVID-19 pandemic has severely impacted mental health across nations. Meta-analytic evidence shows an exacerbation of mental health issues such as depression, anxiety, distress, and insomnia brought about by this unprecedented global health crisis [2].

In light of these findings, there has, however, been a proliferation of research on *psychological resilience* and studies examining how this important psychological ability serves as an important factor in sustaining mental health. Resilience is broadly defined as the ability to bounce back from adversity and crisis. Resilient individuals engage in effective coping that assists them in healthily adjusting to challenges and prevent them from being overwhelmed by adverse events [3]. Recent studies show that resilient individuals are able to maintain their mental health even amidst the COVID-19 pandemic [4, 5].

Psychological resilience can be broadly divided into *protective* and *promotive factors* that help individuals recover from stressful events [6, 7]. Researchers have identified a cluster of variables that serve protective and promotive effects on psychological resilience. These include ego-resilience [8–10], sense of control [11–13], positive emotions [14, 15], grit [16–18], self-compassion [19, 19, 21], and passion [22–24]. The role of psychological resilience in reducing the COVID-19-related fear can be made more apparent by assessing these variables as each distinctly contributing to psychological resilience. Further, it is likely that their relative potency in contributing to an individual’s psychological resilience will vary across contexts.

Furthermore, there are likely cultural differences that influence the degree to which psychological resilience sustains individuals’ mental health. Additional factors—namely, differences in infection status, number of infected people, infection status of family members, and the measures taken by the respective governments should be considered when assessing the underlying psychological drivers of resilience [14]. To understand possible contextual and cultural differences in psychological resilience, we conducted a web survey on these resilience factors, and their effects on mental health from October 2020 to November 2020. We sampled respondents from Japan, China, the United States (US), and Malaysia. This series of research projects is part of the Resilience to CoVid-19 in each region project (RE-COVER Project; Survey on Resilience, Mental Health, and Fear of COVID-19 in Four Countries).

## Data description

The study was approved by the University of Tsukuba’s research ethics. A web survey was conducted from October 14 to November 4, 2020, using a crowdsourcing service (Japan; Crowd Works, US; Amazon Mechanical Turk) and web survey companies (China; Wen Juan Xing, Malaysia; Vase.ai.). Participants provided consent to participate in the survey and were assured that their data would be anonymized and published without identifying information. Participants received a $1-$2 monetary reward upon completion of the survey and took, on average, 25 min to complete the survey.

Data from a total of 1,583 usable responses were collected *(Mean age* = *32.22, SD* = *12.90, Range* = *19–82*). The sample comprised 322 Japanese, 505 Chinese, 333 Americans, and 423 Malaysian respondents. We checked the responses and removed respondents who stated that they were under 18 years old.

We collected a wide range of demographic data, including age, sex, marital status, number of children, occupation, self-isolation status (i.e., extent to which respondents spent time indoors/outdoors), change in income (e.g., income increased), COVID-19 infection status (e.g., family member infected), place of residence, and subjective social status. Several incomplete responses were included in the final dataset given that these were data from respondents who declined to answer some free-text items and/or skipped optional questionnaire items. Even if some of the scales are not answered, other scales can be used in the analysis so that the sample size is not reduced unnecessarily. There were marginally more male respondents than female respondents (*Male* = *931,* Female = 643, Other = 9). In the Chinese dataset, there were more students, and the mean age of respondents was moderately lower (Mean age_*Japan*_ = 38.72, Mean age_*Malaysia*_ = 32.72, Mean age_*China*_ = *21.79,* Mean age_*US*_ = *41.47)*.

Depression, anxiety, and stress were assessed using the 21-item Depression Anxiety Stress Scales (DASS-21) [25–27]. We also assessed for temporal life satisfaction using the 3-item Scale of Present, Past, and Future Life Satisfaction [12, 28] and fear of COVID-19 using the 7-item Fear of COVID-19 Scale [29–31]. Ego resilience was assessed using the 14-item Ego Resiliency Scale [8–10], Sense of Control with the 12-item Sense of Control Scale [11–13], Positive Emotions via the 38-item Dispositional Positive Emotions Scales [14, 15], Grit with the 8-item Grit scale [16–18], Self-compassion with the 26-item Self-Compassion Scale [19–21], and Passion with the 17-item Passion Scale [22–24]. These questionnaires assessed the resilience factors in this study. Finally, we measured relational mobility using the 12-item Relational Mobility Scale [32] as a proxy for cultural differences. The Dispositional Positive Emotions Scales and Scale of Present, Past, and Future Life Satisfaction were translated into Chinese and examined for reliability and validity. For Malaysians, we prepared questionnaires in both English and Malay.

The word file summarizes the scales and demographic descriptions used in this study. In the Excel file, the raw data of the survey and the calculated scores of the scales are summarized [33]. The items for each language of the scale used in this study are described in the source code [33] (Table 1).

**Table 1:** Overview of data files/data sets

Label	Name of data file/data set	File types (file extension)	Data repository and identifier (DOI or accession number)
Data set 1	Data set_RECOVER	MS Excel file (.xlsx)	OSF (https://doi.org/10.17605/OSF.IO/P56GA) [33]
Data file 1	Source code_RECOVER	MS Word file (.doc)	OSF (https://doi.org/10.17605/OSF.IO/P56GA) [33]

## Limitations

Due to the nature of cross-sectional study [33] design, it is not possible to identify a causal relationship between resilience and mental health. In addition, the sample does not include data from other regions in the world such as from Europe, Africa, and South America. As such, we could only compare the results broadly between North America and Asia. It should be noted that this is a cross-sectional data set that only includes data for respondents from October to November 2020. Since the data was obtained through an online survey, which may have caused satisfice, screening the responses would have resulted in a more accurate analysis [34]. The data also do not allow us to determine whether a person has been infected with COVID-19, and as such, we are not able to disentangle whether the effects here are due to actual experiences of being infected, or from perceived threat of infection. These questions remain avenues for further research.

## Data Availability

The data described in this Data note can be freely and openly accessed on Open Science Framework under Identifier: https://doi.org/10.17605/OSF.IO/P56GA. Please see Table 1 for details and links to the data [33].

## Abbreviations

WHO: World Health Organization
COVID-19: Coronavirus disease 2019
RE-COVER: Resilience to CoVid-19 in each region
